# A first approach for the micropropagation of the medicinal halophyte *Polygonum maritimum* L. and phenolic profile of acclimatized plants

**DOI:** 10.3389/fpls.2022.960306

**Published:** 2022-08-30

**Authors:** Luísa Custódio, Sylwester Slusarczyk, Adam Matkowski, Viana Castañeda-Loaiza, Eliana Fernandes, Catarina Pereira, Maria João Rodrigues

**Affiliations:** ^1^Centre of Marine Sciences, Faculty of Sciences and Technology, University of Algarve, Faro, Portugal; ^2^Department of Pharmaceutical Biology and Botany, Wroclaw Medical University, Wroclaw, Poland

**Keywords:** sea knotgrass, salt tolerant plants, plant tissue culture, phenolic compounds, ultra-high-resolution mass spectrometry

## Abstract

*Polygonum maritimum* L. (sea knotgrass) belongs to a genus commonly used in folk medicine to treat inflammation-related disorders. *In vitro* pharmacological studies have confirmed these properties that were ascribed to bioactive flavonoids, such as myricetin and quercetin glycosides. Therefore, this study aimed at establishing a micropropagation procedure for sea knotgrass for obtaining standardized materials for its potential commercial cultivation. For that, a complete plant regeneration protocol was developed by improving shoot multiplication from nodal explants, rooting and acclimatization procedures, followed by the assessment of the phenolic profile of the *in vitro*-produced plants. The combination of 3 mg/L 6-benzylaminopurine (BA) + 0.1 mg/L indole-3-acetic acid (IAA) induced the maximum shoot formation (10.3), which was significantly increased from the first to the second cycle (18.3). The best rooting capacity was observed on shoots derived from the control medium (100%), followed by 2 mg/L kinetin (KIN) (97%) and 3 mg/L BA + 0.1 mg/L IAA (90%); however, the shoot number at the end of the rooting phase was higher on shoots derived from 3 mg/L BA + 0.1 mg/L IAA (6.16). The plant growth regulators used in the multiplication phase influenced survival in the acclimatization process, and plants derived from the control medium had the highest survival percentage (63.1%). Acetone extracts made from aerial organs of micropropagated sea knotgrass showed a predominance of the flavonoid myricetin-3-*O*-rhamnoside (8.135 mg/g). Overall, the halophyte sea knotgrass was successfully micropropagated showing its potential as a medicinal crop for the extraction of bioactive molecules.

## Introduction

One of the main challenges of developing medicinal plants as crops is the high commercial demand for scaling up their cultivation. The most effective way of addressing these needs is by improving plant breeding (Moraes et al., 2021). The amount and quality of the produced bioactive natural compounds are dependent on several factors, including genotype, and thus, plant tissue culture techniques, such as *in vitro* micropropagation, may contribute to obtaining genetically similar plants with standardized contents of bioactive metabolites of selected plants (Espinosa-Leal et al., 2018; Akin, 2020). Such tools allow producing high-yielding clones for industrial applications, running as a nursery for producing stock plants for ensuring the supply of high-scale greenhouse cultivation of selected medicinal plants producing high-quality bioactive ingredients for harmonized commercial products (De Lima et al., 2010; Moraes et al., 2021).

*Polygonum maritimum* L. (common name: sea knotgrass) is a medicinal halophyte belonging to a genus where the aerial parts are commonly used in folk medicine to treat inflammation-related disorders (Bounda and Feng, 2015; Rodrigues et al., 2017, 2018, 2019a,b). Pharmacological studies of different extracts have confirmed that this species holds high *in vitro* antioxidant, anti-inflammatory, neuroprotective, antidiabetic, and anti-hyperpigmentation activities, ascribed to the occurrence of high levels of bioactive flavonoids, including myricetin and quercetin glycosides (Rodrigues et al., 2017, 2018, 2019a). Moreover, this species already revealed high suitability for greenhouse production in saline conditions while preserving its medicinal and chemical properties (Rodrigues et al., 2019b,c), which highlights its potential use as a sustainable source of bioactive ingredients and products for commercial uses.

Despite *in vitro* culture systems being already developed for other non-halophyte *Polygonum* species, including *Polygonum acre* Kunth var. *aquatile* (Mart.) Meisn (De Lima et al., 2010), *Polygonum hydropiper* L. (Hasan and Sikdar, 2010), *Polygonum microcephallum* D.Don. (Das and Handique, 2002), *Polygonum multiflorum* Thunb. (Lin et al., 2003), or *Polygonum glabrum* Willd (Haneef et al., 2019), to the best of our knowledge, there are no reports on micropropagation techniques for improving the multiplication of the sea knotgrass. Therefore, the purpose of this study was to establish a micropropagation procedure for sea knotgrass for obtaining standardized plant materials for the commercial cultivation of this medicinal species. For that, a complete plant regeneration protocol was developed for the first time through the enhancement of shoot multiplication from nodal explants, rooting and acclimatization procedures, followed by the assessment of the metabolic profile of the *in vitro*-produced plants, after the acclimatization phase and after 90 days of greenhouse cultivation.

## Materials and methods

### Chemicals

6-Benzylaminopurine (BA), 1-naphthaleneacetic acid (NAA), indole-3-acetic acid (IAA), kinetin (KIN), Murashige and Skoog (MS) basal medium, and mercury (II) chloride (HgCl_2_), were acquired from Sigma-Aldrich, Germany. Dimethyl sulfoxide, acetone, and additional solvents and reagents were bought from VWR International, Belgium.

### Micropropagation

#### Plant material and explant preparation

*Polygonum maritimum* aerial parts were collected from the Formosa Lagoon (Faro beach, south of Portugal) in February 2020 (coordinates: 36° 59' 57.6528” N 7° 58' 48.9252” W). The aerial parts were washed under running tap water, immersed in tap water with commercial soap for 15 min, and repeatedly rinsed with distilled water. The leaves were removed and discarded, and the stems were divided into nodal segments of 1–2 cm and used as explants.

#### Explant disinfection

Explants (10 per treatment, in three replicates) were surface sterilized by immersion in different solutions (all containing 0.5% Tween20, v/v): (1) 2.5% sodium hypochlorite (NaClO) for 30 min; (2) 2.5% sodium hypochlorite for 30 min followed by 70% ethanol for 10 and (3) 20 min; and (4) 0.5% mercury (II) chloride (HgCl_2_) for 5 and (5) 10 min. Then, explants were rinsed three times with sterile distilled water, as well as between immersion solutions. Afterward, the treatment allowing for the highest disinfection percentage was selected and repeated to obtain the maximum number of sterile explants for the next experiments.

#### Establishment

The explants were placed in individual glass tubes (20 × 2.0 cm) containing 15 ml of MS basal medium (Murashige and Skoog, 1962) free of plant growth regulators (PGRs), with 2% sucrose and 0.8% agar, and maintained under the temperature of 25 ± 2°C, with a 16/8 h light/dark photoperiod provided by LED light (2700 kelvin) at 3,000 lux of light intensity, and then proceed for multiplication. All media were adjusted to pH 5.8 prior autoclaving at 121°C for 20 min.

#### Multiplication and rooting

Shoots generated from disinfected explants were divided into four groups for subcultivation: (1) MS basal medium free of PGRs, (2) MS medium supplemented with 0.06 mg/L of BA and 0.24 mg/L of NAA (De Lima et al., 2010), (3) MS medium supplemented with 3 mg/L of BA and 0.1 mg/L of IAA (Das and Handique, 2002), and (4) MS medium supplemented with 2 mg/L of KIN (Hasan and Sikdar, 2010). Cultures were maintained for 60 days or until enough shoots were available to establish the experiments. The obtained shoots were subjected to a second multiplication cycle. Then, the generated shoots from the different conditions were separated and transferred to MS basal medium free of PGRs for rooting for 60 days. Shoots were counted and the highest shoot measured at the beginning of the multiplication phase and at the end of the rooting phase. All media were adjusted to pH 5.8 prior autoclaving at 121°C for 20 min.

#### Acclimatization

For the *ex vitro* acclimatization, rooted plantlets were transferred to 1-L plastic pots with beach sand as substrate and placed indoors into a transparent lidded plastic box under high humidity (90–100%), natural light (2,500–3,000 lux), and room temperature (25 ± 2°C) conditions for 7–10 days. Plants were progressively adapted to culture room conditions (55–60% humidity, 2,500–3,000 lux, and 25 ± 2°C) by gradually opening the lid from 1 h/day until staying completely open after 30 days. To prevent fungal contaminations, an antifungal commercial solution (difenoconazole 0.003%, v/v) was applied weekly. Then, the plant survival percentage was determined, and plants transferred to the greenhouse for an additional 90 days.

### Chemical composition

#### Plant material and extraction

Samples of sea knotgrass aerial parts were collected at the end of the acclimatization phase and after 90 days of greenhouse cultivation, then dried for 3 days at 40°C, powdered, and stored at −20°C until needed. Then, the dried biomass was extracted with acetone by an ultrasound-assisted procedure (1:40, w/v) for 30 min (ultrasonic bath USC-TH (VWR, Portugal), capacity of 5.4 L, frequency of 45 kHz, supply of 230 V, a tub heater of 400 W, temperature control made by an LED display (Rodrigues et al., 2019a). Extracts were filtered (Whatman no. 4), evaporated under reduced pressure and temperature in a rotary evaporator, weighted, dissolved at appropriate concentration, and stored at −20°C.

#### Phenolic profile by ultra-high-resolution mass spectrometry (UHRMS)

The extracts were diluted to 1 mg/ml using 80% MeOH acidified with 0.2% formic acid. The sample was then centrifuged (23,000 × *g*, 5 min) and filtered (0.22 μm) before LC-MS analysis. All analyses were performed in triplicate for three independent samples (stored at −20°C before analysis for no longer than 3 days). All analyses were performed in triplicate for three independent samples (stored at −20°C before analysis for no longer than 3 days).

Liquid chromatography (LC)–electrospray ionization (ESI) quadrupole time-of-flight (QTOF) MS estimation of polyphenol composition was carried out on a Thermo Dionex Ultimate 3000 RS (Thermo Fischer Scientific, Waltham, MA, USA) chromatographic system, coupled to a Bruker Compact (Bruker, Billerica, MA, USA) QTOF mass spectrometer, consisting of a binary pump system, sample manager, column manager, and PDA detector. Separations were performed on a Kinetex C18 column (2.1 × 100 mm, 2.6 μm, Phenomenex, USA), with mobile phase A consisting of 0.1% (v/v) formic acid in water and mobile phase B consisting of 0.1% (v/v) formic acid in acetonitrile. A linear gradient from 1 to 60% phase B in phase A over 20 min was used to separate phenolic compounds. The flow rate was 0.4 ml/min, and the column was held at 30°C. Spectra were acquired in negative-ion mode over a mass range from *m/z* 100 to 1,500 with 5-Hz frequency. Operating parameters of the ESI ion source were as follows: capillary voltage 3 kV, dry gas flow 6 L/min, dry gas temperature 200°C, nebulizer pressure 0.7 bar, collision radiofrequency 700.0 V, transfer time 100.0 μs, and pre-pulse storage 7.0 μs. Ultrapure nitrogen was used as drying and nebulizer gas, and argon was used as collision gas. The collision energy was set automatically from 15 to 75 eV depending on the *m/z* of the fragmented ion. Acquired data were calibrated internally with sodium formate introduced to the ion source at the beginning and end of each separation *via* a 20-μl loop. Processing of spectra was performed using the Bruker DataAnalysis 4.3 software. The quality of the isotopic fit is expressed by the mSigma-value. The matched peaks from SmartFormula3D were sent to the MetFrag website for computer-assisted *in silico* fragmentation and identification of metabolite mass spectra. Additionally, web-based databases were used to search for the identity of the detected compounds: human metabolome database (http://www.hmdb.ca/), BiGG database (http://bigg.ucsd.edu/), PubChem database (http://pubchem.ncbi.nlm.nih.gov/), MassBank database (http://www.massbank.jp), KEGG (www.genome.jp), and Metlin database (http://metlin.scripps.edu).

After data acquisition, raw UPLC–QTOF-MS spectra (negative mode) were pre-processed using the ProfileAnalysis software (version 2.1, Bruker Daltonik GmbH, Germany). Parameters of ProfileAnalysis were used as follows: advanced bucket generation with retention time range of 0–20 min, mass range of 100–800 *m/z*, each bucket (spectral bins) was formed with 1 min and 1 *m/z* delta, 0.2 kernelizing value, without normalization, background subtraction, and time alignment. LC-MS analyses were processed with the Find Molecular Futures function to create compounds (molecular features) with S/N-3 for peak detection. The generated bucket table consisting of tR:*m/z* pairs and respective compound intensity was exported and uploaded to MetaboAnalyst program.

The detected compounds are expressed quantitatively based on 254-nm peak areas as (-)-epicatechin (CAS 490-46-0) equivalents for flavan-3-ols, chlorogenic acid equivalents (CAS 327-97-9, 3-caffeoylquinic acid) for free phenolic compounds, and isoquercetin equivalents (CAS 482-35-9, quercetin 3-*O*-glucopyranoside) for flavonols. Stock solutions of (-)-epicatechin, chlorogenic acid, and isoquercetin were prepared in MeOH at concentrations of 3.2, 4.1, and 4.5 mg/ml, respectively. Calibration curves for these compounds were constructed based on seven concentration points. After data acquisition, raw UPLC–QTOF-MS spectra (negative mode) were preprocessed using the ProfileAnalysis software (version 2.1, Bruker Daltonik GmbH, Germany).

### Statistical analyses

Results are expressed as mean ± standard error of the mean (SEM), and experiments were led at least in triplicate. Significant differences were evaluated by ANOVA and Tukey's honestly significant difference (HSD) test (*p* < 0.05). Statistical analyses were made using the XLSTAT statistical package for Microsoft Excel (version 2013, Microsoft Corporation).

## Results

### Micropropagation

Appropriate disinfection of the initial explants is essential to developing a successful protocol for *in vitro* propagation. In this work, nodal explants of sea knotgrass collected from the wild were subjected to different sterilization treatments before being cultured on MS media, and new shoots counted after 30 days of *in vitro* culture (Table 1). Among the different treatments tested, 0.5% HgCl_2_ for 5 min allowed for the highest disinfection percentage (50%), whereas explants disinfected with 2.5% NaClO for 30 min had the highest percentage of new shoot formation (20%). The sterilized explants were used for shoot multiplication.

**Table 1:** Percentage of disinfection and shooting of sea knotgrass explants subjected to different sterilization treatments and cultured in MS medium for 30 days (*n* = 30).

**Treatment**	**Disinfection (%)**	**Shoots (%)**
2.5% NaClO; 30 min	16.7	20.0
2.5% NaClO; 30 min + 70% EtOH; 10 min	30.2	6.9
2.5% NaClO; 30 min + 70% EtOH; 20 min	38.5	2.7
0.5% HgCl_2_; 5 min	50.0	6.6
0.5% HgCl_2_; 10 min	26.7	12.5

**Figure 1:**
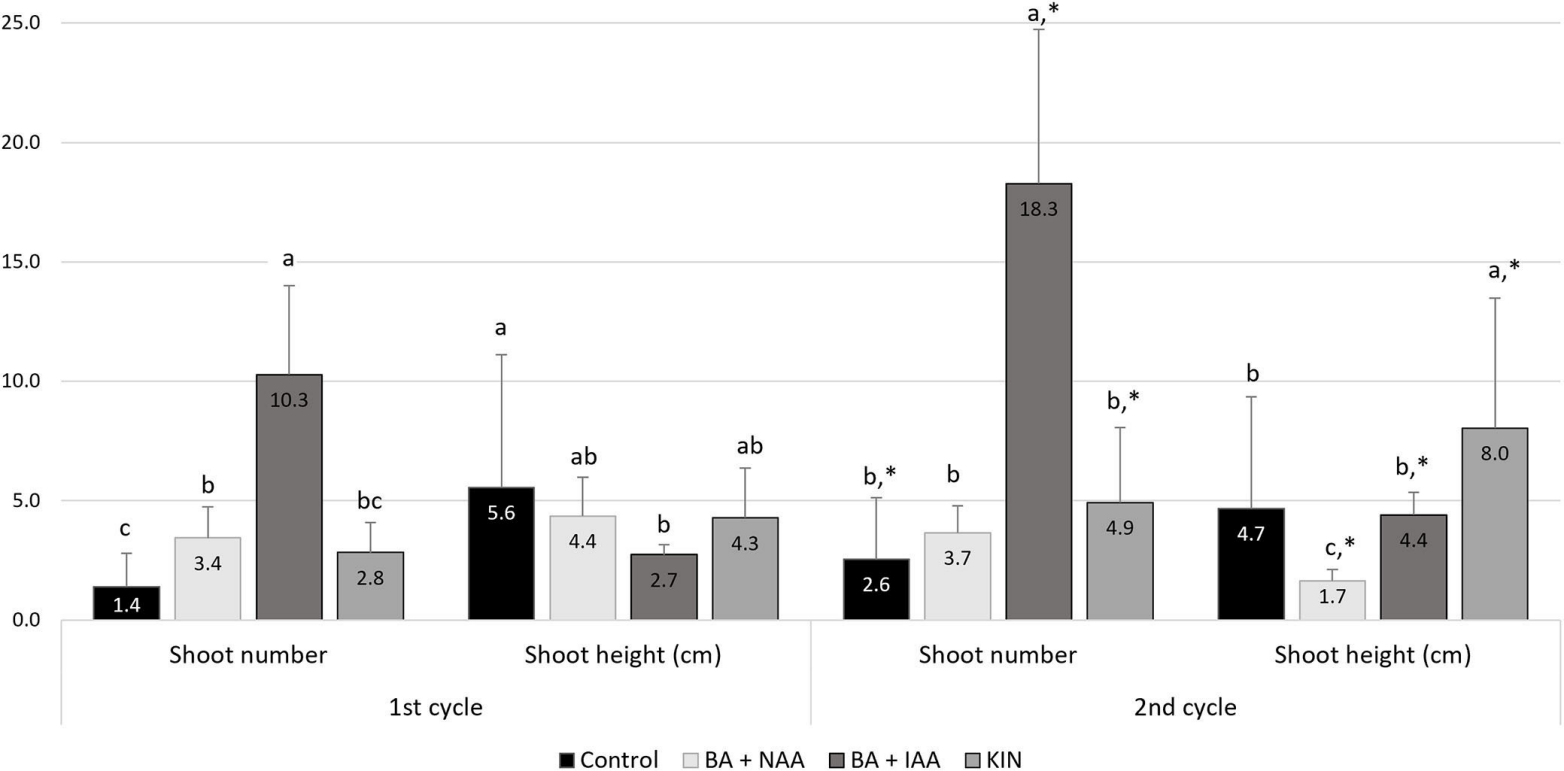
Effect of two consecutive cycles of *in vitro* shoot multiplication of sea knotgrass explants cultivated in MS medium alone (control) and with different growth regulators (0.06 mg/L BA + 0.24 mg/L NAA, 3 mg/L BA + 0.1 mg/L IAA, and 2 mg/L KIN) after 60 days. Values correspond to mean ± SD of three independent experiments (*n* = 30). For each group, columns marked with different letters (a–c) are considered statistically different at *p* < 0.05 (Tukey's HSD), while columns marked with * are significantly different to the respective treatment of the first cycle (*p* < 0.05; Tukey's HSD).

**Figure 2:**
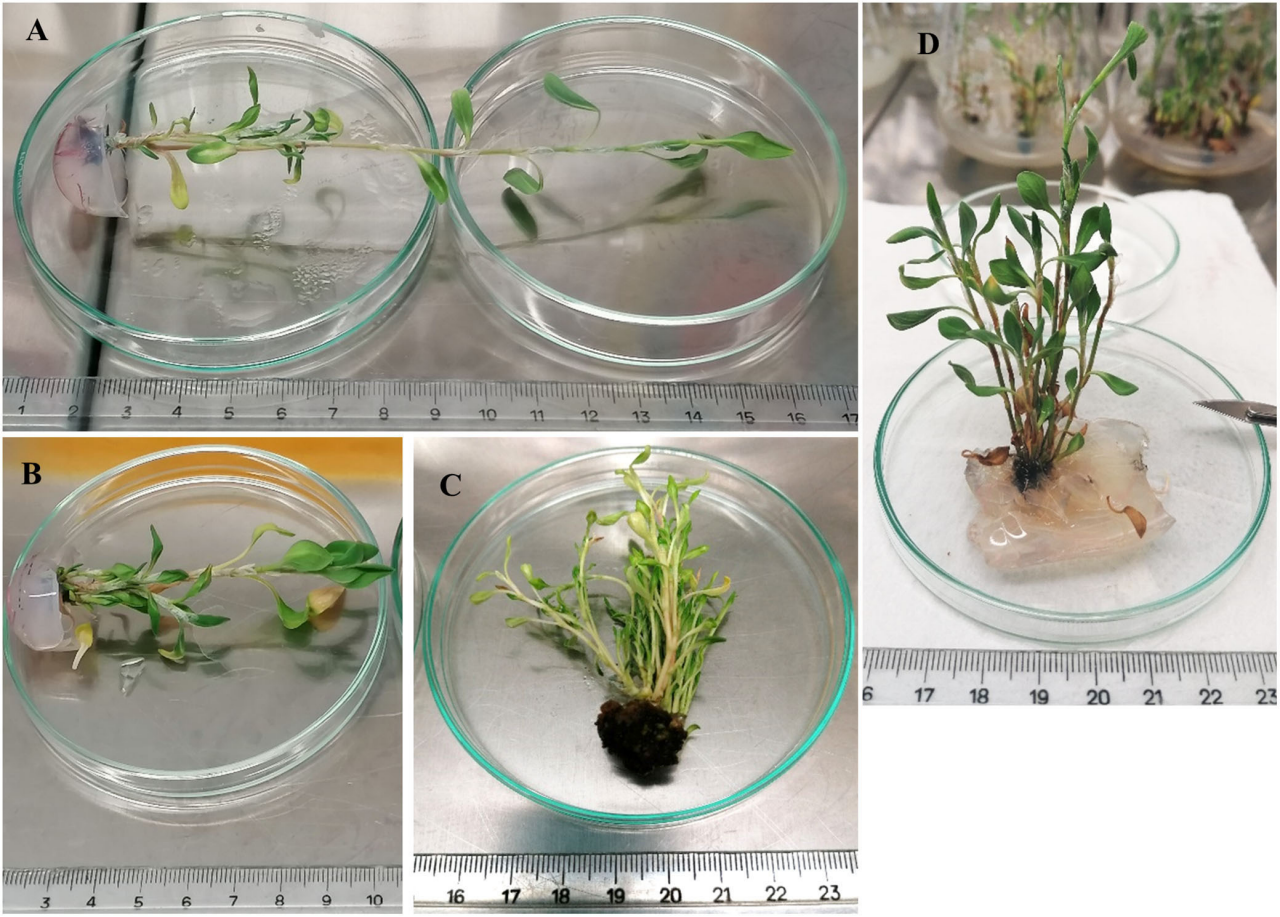
Aspect of 60-day-old sea knotgrass shoots cultivated in MS medium alone **(A)**, 0.06 mg/L BA + 0.24 mg/L NAA **(B)**, 3.0 mg/L BA + 0.1 mg/L IAA **(C)**, and 2.0 mg/L KIN **(D)**.

Nodal explants of sea knotgrass were cultured on MS media alone and supplemented with various combinations of PGRs, namely, 0.06 mg/L BA + 0.24 mg/L NAA, 3 mg/L BA + 0.1 mg/L IAA, and 2 mg/L KIN, and kept for 60 days in two consecutive multiplication cycles. Results are presented in Figure 1 and 60-day-old shoots in Figure 2. In the first cycle, the combination of 3 mg/L BA + 0.1 mg/L IAA induced the maximum shoot formation (10.3 shoot/explant), but the lowest shoot height (2.7 cm). In turn, explants cultured in MS medium without PGRs showed the lowest shoot number (1.4), but the highest shoot height (5.6 cm). The same pattern was observed for the number of shoots at the end of the second multiplication cycle, and the treatment consisting of 3 mg/L BA + 0.1 mg/L IAA significantly increased the shoot formation (18.3 shoot/explant), while 2 mg/L KIN improved the height of produced shoots (*p* < 0.05). Also, in exception to control plants for the first cycle, all the treatments induced the growth of compact dark green callus (first cycle: 94.4–100%; second cycle 20.6–100%; Table 2), and explants from all the conditions developed roots (first cycle: 23.1–88.9%; second cycle 11.1–33.3%), excluding the combination of 3 mg/L BA + 0.1 mg/L IAA (Table 2).

**Table 2:** Percentage of callus formation and rooting of sea knotgrass explants subjected to two consecutive cycles of *in vitro* shoot multiplication cultivated in MS medium alone (control) and with different growth regulators (BA + NAA; BA + IAA and KIN) after 60 days.

	**First cycle**		**Second cycle**	
**Treatment**	**Callus (%)**	**Rooting (%)**	**Callus (%)**	**Rooting (%)**
Control	0	57.1	20.6	26.5
0.06 mg/L BA + 0.24 mg/L NAA	94.4	88.9	69.4	11.1
3 mg/L BA + 0.1 mg/L IAA	100	0	100	0
2 mg/L KIN	100	23.1	100	33.3

**Figure 3:**
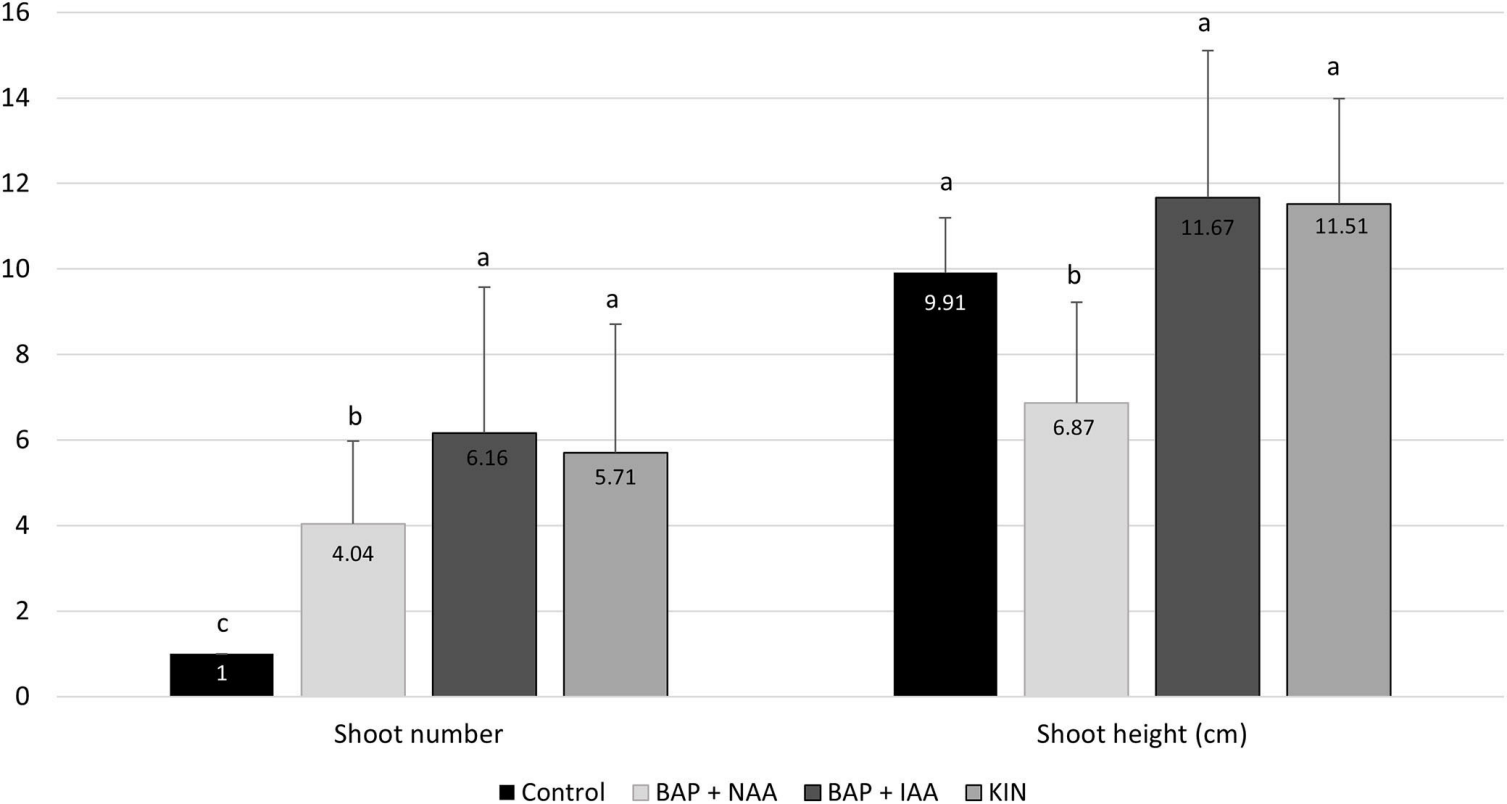
Effect of the *in vitro* rooting phase on the shoot number and height of the sea knotgrass cultivated in MS medium derived from the different multiplication media (control, 0.06 mg/L BA + 0.24 mg/L NAA, 3.0 mg/L BA + 0.1 mg/L IAA, and 2.0 mg/L KIN) after 60 days. Values correspond to mean ± SD of three independent experiments (*n* = 30). Columns marked with different letters (a–c) are considered statistically different at *p* < 0.05 (Tukey's HSD).

In the rooting phase, shoots obtained from the different treatments were cultivated in MS medium free of PGRs for 60 days, and results are presented in Table 3. All the shoots derived from the control medium were able to develop roots (100%), followed by shoots obtained from MS medium supplemented with 2 mg/L KIN (97%) and from 3 mg/L BA + 0.1 mg/L IAA (90%), while 0.06 mg/L BA + 0.24 mg/L NAA-derived shoots presented the lowest rooting capacity (54.9%). The effect of the *in vitro* rooting phase on the shoot number and height was also assessed (Figure 3). Shoot number was highest on shoots derived from 3 mg/L BA + 0.1 mg/L IAA (6.16 shoots/plantlet) and lowest on shoots from the control treatment (1 shoot/plantlet); however, the shoot height was similar among control, 3 mg/L BA + 0.1 mg/L IAA, and 2 mg/L KIN (9.91, 11.67, and 11.51 cm, respectively), while 0.06 mg/L BA + 0.24 mg/L NAA-derived shoots had the lowest height.

**Table 3:** Percentage of rooting of sea knotgrass shoots cultivated in MS medium, obtained from different multiplication media, after 60 days.

**Multiplication media**	**Rooting (%)**
Control	100
0.06 mg/L BA + 0.24 mg/L NAA	54.9
3 mg/L BA + 0.1 mg/L IAA	90
2 mg/L KIN	97

Then, rooted plantlets were transferred to plastic trays containing beach sand as substrate and were progressively acclimatized to culture room conditions using lidded plastic boxes that were gradually opened from 1 h/day until staying completely open after 30 days. The plant survival percentage was then determined and is shown in Table 4. The PGRs used in the multiplication phase influenced their survival in the acclimatization process. Plants multiplied in the control medium (without growth regulators) had the highest survival percentage (63.1%), followed by those from medium supplemented with 2 mg/L KIN (53.6%), 3 mg/L BA + 0.1 mg/L IAA (45.1%), and finally with the lowest survival rate by 0.06 mg/L BA + 0.24 mg/L NAA (23.5%).

**Table 4:** Percentage of survival of sea knotgrass plants after 30 days of acclimatization.

**Treatment**	**Survival (%)**
Control	63.1
0.06 mg/L BA + 0.24 mg/L NAA	23.5
3 mg/L BA + 0.1 mg/L IAA	45.1
2 mg/L KIN	53.6

### Chemical composition

The acetone extracts of leaves collected from sea knotgrass after 30 days of acclimatization and after 90 days of greenhouse cultivation were analyzed by LC-MS for the detection of major phenolic compounds (Table 5). The obtained data revealed a predominance of one compound—myricetin-3-*O*-rhamnoside (8.135 mg/g), which constituted approximately half of all phenolics in the micropropagated sea knotgrass. Other myricetin glycosides were also detected as minor constituents, namely, myricetin-diacetylrhamnoside (1.411 mg/g). The quercetin glycosides (quercetin-3-*O*-galactoside and quercitrin) occurred in lower amounts (0.833 and 1.053 mg/g, respectively). The second class of phenolics present in higher abundance were hydroxybenzoic acids (gallic acid and tentatively identified gallic acid-*O*-glucoside), which constituted ~20% of the sum of the compounds (3.633 mg/g). Other tentatively identified compounds included coumaric acid and a dicarboxylic aliphatic acid (azelaic acid). In turn, the composition of the sea knotgrass after 90 days of greenhouse cultivation was completely different. The major compounds were various derivatives of hydroxycinnamic acids (mainly glucosides), including caffeic acid and its glucosides that occurred as three isomers constituting almost 30% of summed phenolics (15.48 mg/g). Catechin (a flavan-3-ol) was the only identified flavonoid and occurred as a minor compound (0.72 mg/g).

**Table 5:** LC-MS/MS analysis with tentative annotation of major phenolic compounds in *P. maritimum* after 30 days of acclimatization **(A)** and after 90 days of greenhouse cultivation **(B)**.

**Compounds**	**RT**	**UV**	**m/z[M-H] -**	**Formula**	**MS^2^ main_ion**	**MS^2^ fragments**	**mg/g**
**(A)**
Galloyl- -glucose	1		331.0667	C_13_H_16_O_10_	169.0133	271,151,125	1.657
Gallic acid	1.2	213	169.014	C_7_H_6_O_5_	125.0235		1.976
ND	2.6		293.1239	C_12_H_22_O_8_	131.071		0.743
*p-*Coumaric acid	7.9		163.0392	C_9_H_8_O_3_	119.0493		0.889
Myricetin 3-O-rhamnoside	9.9	218,263,351	463.0884	C_21_H_20_O_12_	316.0227	271,179	8.135
Quercetin-3-O-galactoside	10.4	262, 355	463.0867	C_21_H_20_O_12_	300.0276	271,179	0.833
Azelaic acid	10.9		187.0977	C_9_H_16_O_4_	125.0966	169.0867	0.749
Quercitrin	11.3	272, 348	447.0923	C_21_H_20_O_11_	300.0272	284,255	1.053
Myricetin -diacetylrhamnoside isomer 1	14		547.1084	C_25_H_24_O_14_	316.0233		0.723
Myricetin -diacetylrhamnoside isomer 2	14.4		547.1078	C_25_H_24_O_14_	316.0217		0.688
**Total**							*17.446*
**(B)**
Mannitol	0.65		181.0718	C_6_H_14_O_6_	163.0601		1.57
Citric acid	0.9		191.0196	C_6_H_8_O_7_	155.111		1.58
Caffeoyl-beta-D-glucose isomer 1	1.4	246,295,325	341.0888	C_15_H_18_O_9_	161.0233		2.26
Glucosyringic acid	1.6	264	359.0986	C_15_H_20_O_10_	197.0453		1.05
3,4-Dihydromethylcatalpol	1.7	264	377.1459	C_16_H_26_O_10_	263.08		2.16
Caffeoyl-beta-D-glucose isomer 2	1.8	246,295,325	341.0888	C_15_H_18_O_9_	161.0233	179,135	0.93
Caffeoyl-beta-D-glucose isomer 3	2.1	246,295,325	341.0888	C_15_H_18_O_9_	161.0233	179,135	8.29
Glucopyranosyl-2-O-methylphloroacetophenone	2.6		343.1051	C_15_H_20_O_9_	161.0237	179,135	5.95
Compound not identified	3.2		377.1462		359.1351	341,179,135,221	0.89
Catechin	3.5	275	289.072	C_15_H_14_O_6_	245.0803	203,221,151,137,125	0.72
Hydroxycinnamic acid-glucoside	3.6	279	325.0933	C_15_H_18_O_8_	145.0284	163,187	0.66
Caffeic acid	4.8	244,292,322	179.0348	C_9_H_8_O_4_	135.044		4.00
Feruloyl-D-glucose	5.2		355.1036	C_16_H_20_O_9_	175.0388	160.015	0.69
An hydroxybenzoic acid derivative	5.5		340.1052	C_15_H_19_N_1_O_8_	161.0447		1.10
alpha-L-rhamnopyranoside				
Compound not identified	6.8		405.1412		179.0702	135	1.00
*p-*coumaric acid	7.7		163.039	C_9_H_8_O_3_	119.0493		0.79
Dicaffeoyl glucose isomer 1	9.9	218,325	503.1199	C_24_H_24_O_12_	179.0344	341,161,135,281	2.37
Dicaffeoyl glucose isomer 2	10.2	218,325	503.1201	C_24_H_24_O_12_	179.0344	341,161,135,281	2.19
Methyl 3,4-dihydroxycinnamate	11		193.05	C_10_H_10_O_4_	161.0221	134	0.74
**Total**							*38.96*

## Discussion

### Micropropagation

*Polygonum* plants are used in folk medicine due to their capacity to synthesize metabolites with biological properties relevant to treating human diseases, mainly those related to inflammatory conditions (Bounda and Feng, 2015; Rodrigues et al., 2017, 2018, 2019a). However, the levels of secondary metabolites are influenced by both genetic and abiotic conditions, such as plant organ, location, and season, which impact the produced level and quality of secondary metabolites, as well as the efficacy of the derived products (Yang et al., 2018; Li et al., 2020). Thus, the high-scale production of medicinal species requires a controlled production allowing for an enhanced metabolites production for obtaining homogenous products for potential commercial uses, which can be achieved by employing *in vitro* propagation techniques (Espinosa-Leal et al., 2018; Akin, 2020). In this context, this work aimed to develop an efficient micropropagation protocol for sea knotgrass and assess the influence of the micropropagation process on the chemical composition of the produced plants.

A similar work on the micropropagation of halophytes did not report the testing of different disinfection methods, but in most studies of other glycophytic *Polygonum* species the most used sterilization agent is HgCl_2_ (0.1–0.5%) between 1 and 10 min (Das and Handique, 2002; Hasan and Sikdar, 2010; Haneef et al., 2019), which is, according to our results, the most effective treatment. Moreover, several *Polygonum* species have been produced by *in vitro* techniques for improved shoot multiplication, but just a few aimed at the production of bioactive metabolites for commercial applications. For example, a successful micropropagation protocol was established for *P. acre* Kunth var. *aquatile* (Mart.) Mesisn, and among the different growth regulators tested, the combination of 0.06 mg/L BA + 0.24 mg/L NAA promoted the highest development of shoots and elongation (2.82 per explant), and no treatment was necessary for rooting (De Lima et al., 2010). Also, Hasan and Sikdar (2010) obtained the highest shoot development of *P. hydropiper* L. (9.0 per explant) when they were cultivated in MS medium supplemented with 2.0 mg/L KIN (Hasan and Sikdar, 2010). In turn, *P. microcephallum* D. Don. explants showed the maximum shoot induction when cultivated in MS medium supplemented with 3.0 mg/L of BA + 0.1 mg/L IAA (4.7 per explant; Das and Handique, 2002). Thus, those conditions were chosen to be tested in this work but in contrast to previous reports, the combination of 3.0 mg/L BA + 0.1 mg/L IAA allowed for the highest shoot number (10.3 in the first cycle). In contrast, in spite of sea knotgrass explants cultivated with MS supplemented with 2.0 mg/L KIN having a lower shoot development, it increased 1.75 times after the second multiplication cycle. Regarding the rooting phase, similar to that observed for *P. acre* (De Lima et al., 2010), for sea knotgrass no treatment was needed for rooting and plantlets previously cultivated in MS medium supplemented with 3.0 mg/L BA + 0.1 mg/L IAA and 2.0 mg/L KIN easily developed roots (90–97% of rooting), when transferred into MS medium without PGRs, comparable to the rooting percentage obtained in plants from medium without PGRs (100%). Additionally, at the end of the rooting phase, the individualized shoots derived from both above-mentioned media produced a similar number of shoots with identical height (Figure 3).

**Figure 4:**
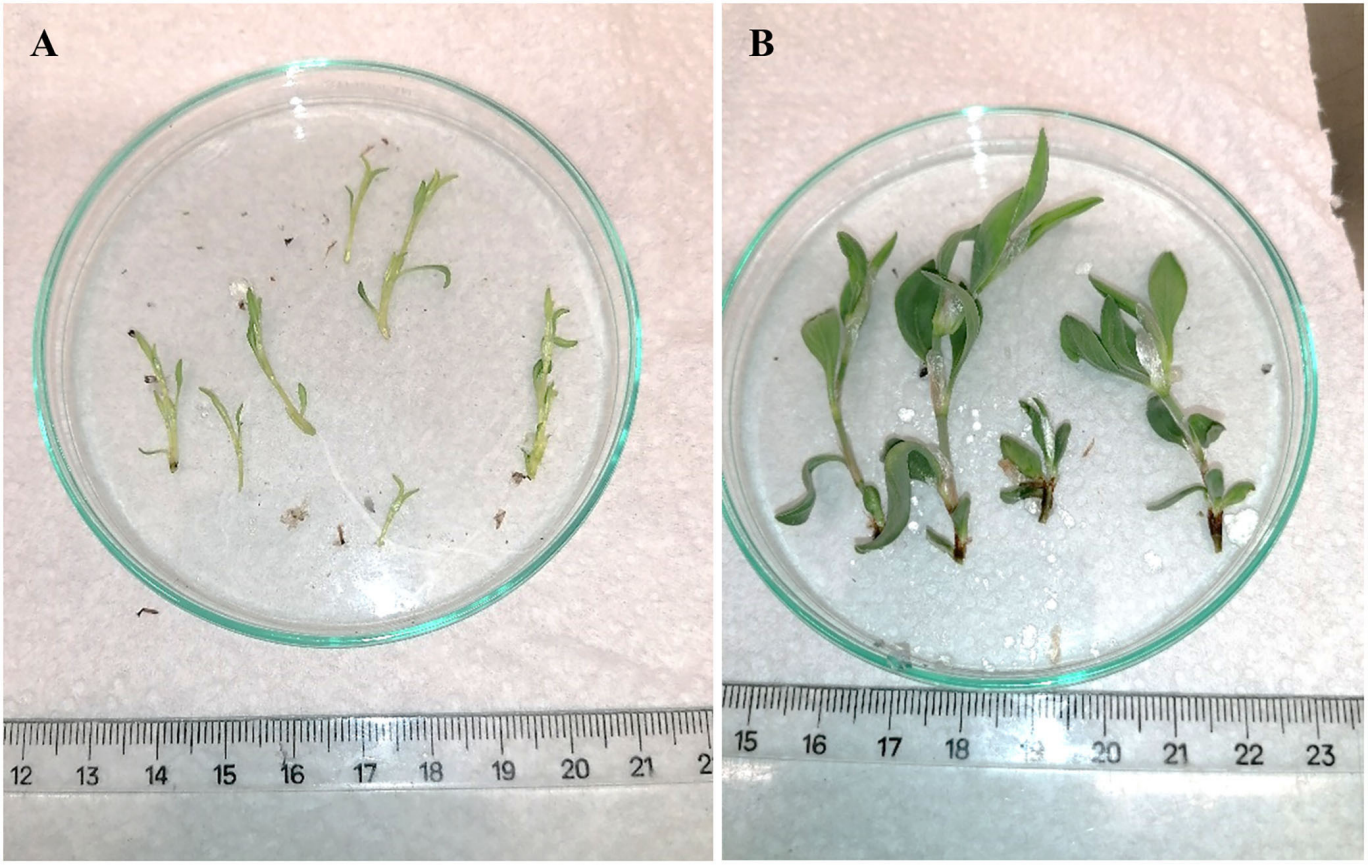
Details of 60-day-old sea knotgrass shoots cultivated in MS supplemented with 3.0 mg/L BA + 0.1 mg/L IAA **(A)** and 2.0 mg/L KIN **(B)**.

The success of *in vitro* propagation depends ultimately on the successful establishment of plants in the soil. Also, as the leaves of *in vitro*-produced plantlets contain minimal cuticular wax and low- or nonactive stomatal systems, they are subjected to high water losses through the leaf surface (Din et al., 2020). Thus, the limited survival of plantlets from MS medium supplemented with 3.0 mg/L BA + 0.1 mg/L IAA can be related to their very tender structure with low developed leaves and fragile stems (Figure 4A) that allows for faster dehydration and consequent death. In turn, plantlets from culture medium with 2.0 mg/L KIN were more robust, presenting more developed leaves and trickier stems (Figure 4B), which were more resistant to the acclimatization process showing the highest survival (53.6%) among the treatments. A strategy to improve the plant survival during the acclimatization phase could be rooting in half-strength MS basal medium since the nutrient and osmotic stresses promote the root formation, as well as the reduction of osmotic potential for improved water uptake, which may result in higher success in the acclimatization of plantlets (Argentel-Martínez et al., 2019).

Overall, the micropropagated sea knotgrass plantlets were obtained by shoot formation from nodal segments with no buds, and the plantlets were derived from new vegetative buds with no signs of morphological alterations that were repeatedly multiplied, which is especially important for micropropagation of medicinal plants, allowing for products standardization and batch-to-batch consistency. Moreover, micropropagation of medicinal species, such as the sea knotgrass, is crucial for scaling up the commercial production once it confers major advantages over conventional propagation techniques, namely, disease-free plantlets, enhanced multiplication rates, contributing to produce higher quantities of homogenous plants without seasonal constraints (Moraes et al., 2021). As such, plant tissue culture is also a major platform for a stable secondary metabolite production, enabling to manipulate the culture conditions to enhance the synthesis of high-quality metabolites of commercial interest in a short time (Espinosa-Leal et al., 2018).

**Figure 5:**
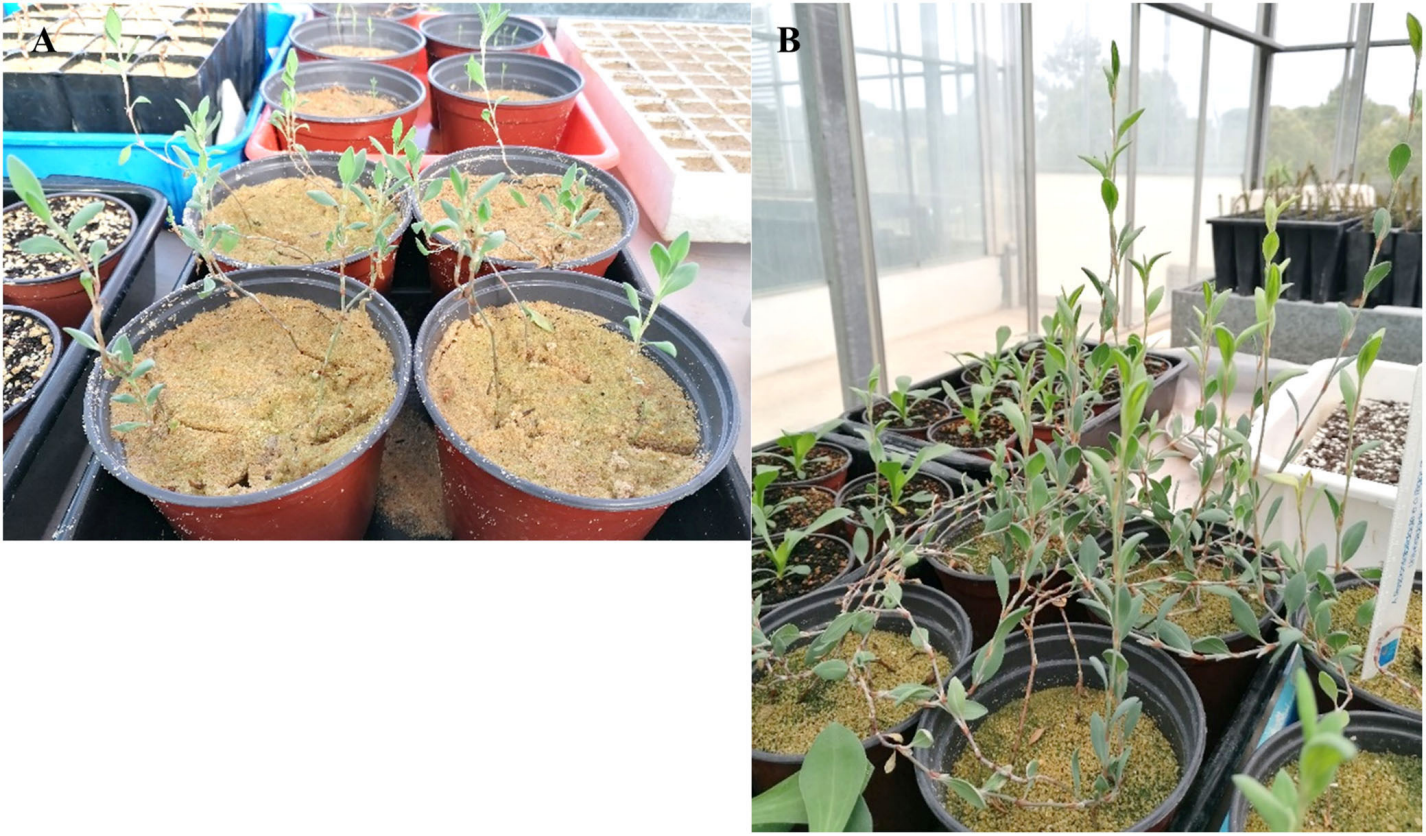
Sea knotgrass plants after 30 days of acclimatization **(A)** and after 90 days in the greenhouse **(B)**.

To ensure that metabolite production were not related to the growth regulators used in the multiplication stage, only plantlets obtained from non-supplemented MS medium (control) were used for subsequent cultivation in the greenhouse (Figures 5A,B) and extraction of metabolites.

### Chemical composition

Sea knotgrass has been identified as a rich source of bioactive phenolic compounds with *in vitro* antioxidant, anti-inflammatory, antimicrobial, antidiabetic, neuroprotective, and anti-melanogenic properties (El-Haci et al., 2013; Rodrigues et al., 2017, 2018, 2019a,b). However, the phenolic composition depicted in this study is distinct from the typical sea knotgrass phytochemistry observed in our previous studies (Rodrigues et al., 2018, 2019a,b). For instance, acetone extracts from plants collected from the wild and grown in a greenhouse under saline conditions presented a higher chemical diversity than those multiplied *in vitro*, namely, 31–47 vs. 10–13 compounds were detected, respectively. Moreover, in wild material there is a marked predominance of myricitrin and catechin, whereas in plants grown in the greenhouse a more considerable presence of catechin was found with a larger proportion of quercetin glycosides (Rodrigues et al., 2018, 2019a,b). Similarly, *in vitro*-produced plants showed a predominance of myricitrin, and other minor myricetin and quercetin glycosides. However, the micropropagated plants were grown in a greenhouse for 90 days; the major constituents detected were hydroxycinnamic acids derivatives (mainly caffeic acid glucosides). Although a slightly different phytochemical profile was observed in this study, it is expected that *in vitro*-produced plants retain the capacity to synthesize the key bioactive molecules, and thus, the biological activities previously reported for this species can be maintained (Rodrigues et al., 2017, 2018, 2019a,b). Moreover, myricetin and its glycosides, the main compounds detected in *in vitro*-produced sea knotgrass, as well as hydroxycinnamic acids, key constituents of plants grown in a greenhouse for 90 days, were described with several pharmacological properties, including high antioxidant capacity, which also gives them a high potential to prevent and alleviate oxidative stress-related diseases such as cancer, diabetes, and cardiovascular diseases, as well as antiviral, antibacterial, anti-inflammatory, analgesic, and hepatoprotective properties (Sova and Saso, 2020; Xu et al., 2020). This highlights the potential use of micropropagated sea knotgrass as a source of bioactive molecules for pharmacological applications. Phenolic compounds are plant secondary metabolites produced by diverse energy-generating routes in metabolic pathways, such as photosynthesis, glycolysis, and Krebs cycle to biosynthetic intermediates. These metabolites are not essential for plant growth and development but have important roles as signaling and defense molecules in response to environmental stimuli, such as climatic fluctuations, pathogenic organisms, predatory herbivores, as well as competing plants. Their synthesis depends on biotic and abiotic conditions, such as growth and physiology, temperature, humidity, and light intensity; therefore, the metabolite production by *in vitro* cultures is dependent on culture conditions. In this sense, since the micropropagated sea knotgrass plants were grown under controlled and constant medium, light (3,000 lux), temperature (±25°C), and pathogen-free conditions, it is thus expectable that their secondary metabolite production of phenolic compounds is lower than that in the plants grown in the greenhouse that were subjected to a higher temperature variation (*T*_min._ ± 10°C, *T*_max._ ± 45°C), as well as higher maximum light intensity (10,000–22,000 lux), and possible microbiological and/or fungal infections, for example. In addition, wild-derived sea knotgrass plants are exposed to more stressful conditions, such as high salinity, light intensity, UV radiation, and/or herbivory, which are factors known to promote plant secondary metabolism. Therefore, it can explain the difference found between the previous studies made with sea knotgrass plants collected from the wild or cultivated from seeds gathered from natural sites, and this study with micropropagated plants. However, the *in vitro* production of secondary metabolites can be stimulated by the addition of biotic (e.g., proteins, fungus, rhizobacteria, and hormones) and/or abiotic (e.g., drought, salinity, light, and temperature) elements to the culture medium to enhance the biosynthesis and accumulation of secondary compounds of interest (Kandoudi and Németh-Zámboriné, 2022). In contrast, for the consequent greenhouse cultivation, growth conditions can also be managed to promote the synthesis of the desired compounds. For instance, low fertilization levels and/or light quality may be negatively correlated to phenolics and flavonoid production (Ibrahim et al., 2012; Alsina et al., 2022). Trials are thus being conducted to understand how the cultivation conditions can be controlled to promote the synthesis of the flavonoids typically produced by this species.

Overall, the micropropagation of the sea knotgrass showed to be a promising way for a rapid and improved multiplication of this species, which can be used as a nursery to supply a high-scale production, contributing to developing it as a medicinal crop.

## Conclusion

The combination of 3 mg/L BA + 0.1 mg/L IAA induced the highest number of new shoots, and plants grown on MS medium free of PGRs had the highest rooting capacity and highest survival during acclimatization. In spite of this, the plants grown with 2.0 mg/L KIN were coupled to a good multiplication with high survival to acclimatization. The flavonoid myricetin-3-*O*-rhamnoside has been identified as the major constituent of the *in vitro*-produced plants, described with several biological activities (e.g., antioxidant, anti-inflammatory). Overall, the sea knotgrass was successfully micropropagated showing its potential as a medicinal crop for the extraction of bioactive molecules for pharmacological applications.

## Data availability statement

The raw data supporting the conclusions of this article will be made available by the authors, without undue reservation. The LC-MS data presented in the study are deposited in the following link https://cloud.umw.edu.pl/index.php/s/bFzNSkWXtsTNYJW.

## Author contributions

LC and MR contributed to conception, design of the study, and wrote the first draft of the manuscript. LC, MR, VC-L, EF, CP, and SS conducted the experimental trials. AM wrote one section of the manuscript. All authors contributed to the article and approved the submitted version.

## Funding

This research was funded by the Foundation for Science and Technology (FCT) and the Portuguese National Budget through projects UIDB/04326/2020 and UID/DTP/04138/2020. It also received funding through Fundo Azul (XtremeAquaCrops project: FA-05-2017-028), and the project HaloFarMs, which is part of the Partnership on Research and Innovation in the Mediterranean Area (PRIMA) program supported by the European Union and funded by the national funding bodies of Participating States (FCT in Portugal). MR was supported through the FCT program contract (UIDP/04326/2020) and LC by the FCT Scientific Employment Stimulus (CEECIND/00425/2017). VC-L and EF acknowledge FCT for PhD grants with the references 2020.04541.BD and UI/BD/151301/2121, respectively. The chemical analysis was supported by the Wroclaw Medical University project no. SUBZ.D030.22.017 (to SS and AM).

## Conflict of interest

The authors declare that the research was conducted in the absence of any commercial or financial relationships that could be construed as a potential conflict of interest.

## Publisher's note

All claims expressed in this article are solely those of the authors and do not necessarily represent those of their affiliated organizations, or those of the publisher, the editors and the reviewers. Any product that may be evaluated in this article, or claim that may be made by its manufacturer, is not guaranteed or endorsed by the publisher.
